# Highlight—Blind as a Bat? The Genetic Basis of Echolocation in Bats and Whales

**DOI:** 10.1093/gbe/evaa003

**Published:** 2020-01-27

**Authors:** Casey McGrath

**FIG. 1.**
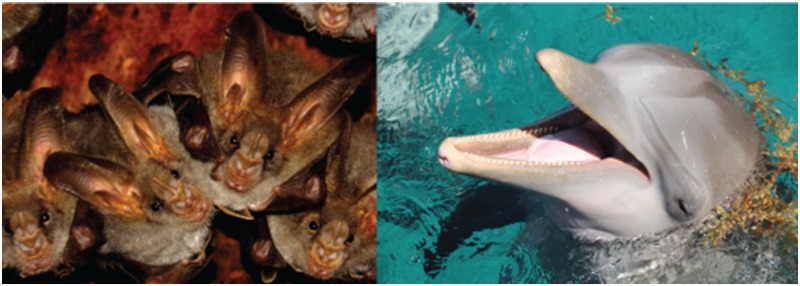
—Many species of bat and toothed whales (including dolphins) evolved echolocation independently. Credit: Vishu Vishuma (left) and Darin Ashby (right).

Clicks, squeaks, chirps, buzzes, etc. though they may be difficult to distinguish to our ears, such sounds are used by echolocating animals to paint a vivid picture of their surroundings. By generating a sound and then listening to how the sound waves bounce off of objects around them, these animals are able to “see” using sound. Although a number of species engage in some form of echolocation, including some birds, shrews, and even humans, the echolocation systems of bats and toothed whales (including dolphins, porpoises, killer whales, and sperm whales) are exquisitely sophisticated. Echolocation evolved independently in these animals (fig. 1) under conditions of poor visibility—the night sky for bats and deep underwater for toothed whales—enabling them to hunt for prey and navigate in complete darkness. It is a fascinating example of convergent evolution, the process by which distantly related organisms evolve similar features or adaptations. To better understand how echolocation evolved in these species, a new study in *Genome Biology and Evolution*, titled “Evolutionary basis of high-frequency hearing in the cochleae of echolocators revealed by comparative genomics,” takes advantage of advances in genomic analysis to investigate the origin and evolution of high-frequency hearing, an adaptation that allows echolocators to perceive ultrasonic signals.

The interpretation of sound begins in the cochlea, a hollow, spiral-shaped bone in the inner ear that converts sound vibrations into nerve impulses. Because gaining the ability to hear very high frequencies likely required changes to the cochlea, the authors of the study, led by Keping Sun and Jiang Feng from Northeast Normal University and Jilin Agricultural University in China, analyzed the genes expressed in the cochleae of three bat species that use different forms of echolocation: constant-frequency, frequency-modulated, and tongue-click echolocation (Wang H, Zhao H, Sun K, Huang X, Jin L, Feng J, in preparation). They then compared these gene sequences to those from 16 other mammals, including other echolocating and nonecholocating bats, as well as both echolocating and nonecholocating whales. According to Sun and Feng, this allowed them to “provide for the first time a comprehensive understanding of the genetic basis underlying high-frequency hearing in the cochleae of echolocating bats and whales.”

Through their comparative analysis, the researchers identified 34 genes involved in hearing or auditory perception that showed evidence for positive selection in echolocating species. This included 12 genes involved in bone formation that may help regulate the bone density of the cochlea to enable high-frequency hearing. It also included several genes with antioxidant activity that may help protect the ear from damage and hearing loss caused by chronic exposure to high-intensity noise. (Bat calls can reach 120 decibels, louder than a rock concert and above the human pain threshold. It is lucky for us that they are too high-pitched for us to hear.)

**FIG. 2.**
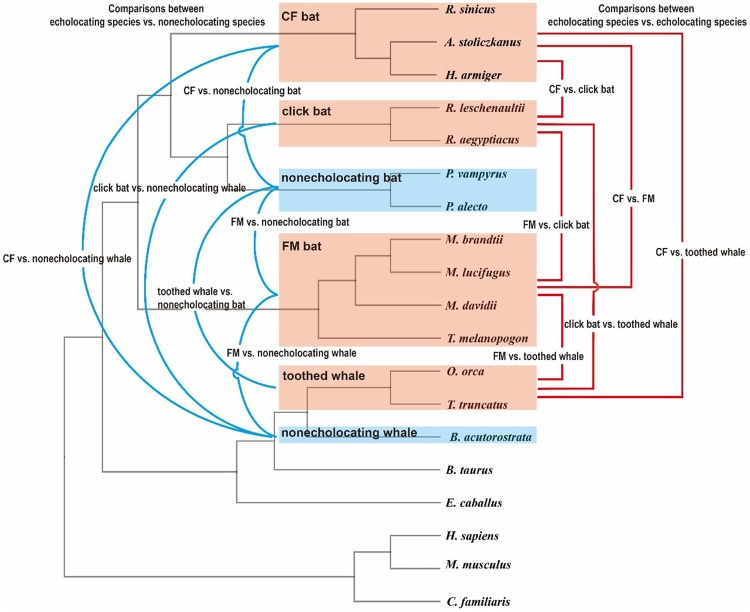
—Pairwise comparisons of parallel/convergent mutations. Red lines on the right represent comparisons between pairs of echolocating species; blue lines on the left represent comparisons between an echolocating species and a nonecholocating species. Echolocating species are highlighted in red and nonecholocating species in blue. CF, constant-frequency bat; FM, frequency-modulated bat. Credit: Keping Sun and Jiang Feng.

The study also revealed large numbers of parallel or convergent mutations between pairs of echolocating mammals, where the same genetic changes had occurred independently in distantly related echolocators. Interestingly, there were significantly more of these parallel/convergent mutations between pairs of echolocators than when comparing an echolocator to an equally distant nonecholocator (fig. 2). This suggests that some of these mutations may play a key role in high-frequency hearing and echolocation.

As noted by Sun and Feng, confirming such a hypothesis requires functional assays for each candidate gene that may underlie echolocation, such as suppressing the expression of hearing genes using RNA interference technology. Unfortunately, such experiments can be costly and both time- and labor-intensive (and in the case of whales, nearly impossible to implement). Despite the remaining challenges, however, the authors are grateful that comprehensive studies like theirs are now possible thanks to recent advances in genomic technologies. “This is an exciting time for the study of adaptive evolution of echolocation in mammals. More and more genetic and genomic data sets have been published, providing insights into the evolutionary basis of echolocation.”
